# Observed and Potential Impacts of the COVID-19 Pandemic on the Environment

**DOI:** 10.3390/ijerph17114140

**Published:** 2020-06-10

**Authors:** Sorin Cheval, Cristian Mihai Adamescu, Teodoro Georgiadis, Mathew Herrnegger, Adrian Piticar, David R. Legates

**Affiliations:** 1 “Henri Coandă” Air Force Academy, 500183 Brașov, Romania; sorin.cheval@afahc.ro (S.C.); adrian.piticar@afahc.ro (A.P.); 2 National Meteorological Administration, 013686 Bucharest, Romania; 3 Research Center for Systems Ecology and Sustainability, University of Bucharest, 050095 Bucharest, Romania; 4 Institute for the BioEconomy-CNR, 40129 Bologna, Italy; teodoro.georgiadis@ibe.cnr.it; 5 Institute for Hydrology and Water Management, University of Natural Resources and Life Sciences (BOKU), 1190 Vienna, Austria; mathew.herrnegger@boku.ac.at; 6 Department of Geography and Spatial Sciences, University of Delaware, Newark, DE 19716-2541, USA; legates@udel.edu; 7 Department of Applied Economics and Statistics, University of Delaware, Newark, DE 19716-2541, USA

**Keywords:** COVID-19, SARS-CoV-2, global environment, pandemic, climate, sustainability

## Abstract

Various environmental factors influence the outbreak and spread of epidemic or even pandemic events which, in turn, may cause feedbacks on the environment. The novel coronavirus disease (COVID-19) was declared a pandemic on 13 March 2020 and its rapid onset, spatial extent and complex consequences make it a once-in-a-century global disaster. Most countries responded by social distancing measures and severely diminished economic and other activities. Consequently, by the end of April 2020, the COVID-19 pandemic has led to numerous environmental impacts, both positive such as enhanced air and water quality in urban areas, and negative, such as shoreline pollution due to the disposal of sanitary consumables. This study presents an early overview of the observed and potential impacts of the COVID-19 on the environment. We argue that the effects of COVID-19 are determined mainly by anthropogenic factors which are becoming obvious as human activity diminishes across the planet, and the impacts on cities and public health will be continued in the coming years.

## 1. Introduction

The Earth is a dynamically changing planet, permanently shaped by socio-ecological interactions. Variations and changes are common in a nonlinear and dynamic system such as our planet but passing certain thresholds may push the stability of the systems into a new regime which can have significant consequences at different spatial and temporal scales. Understanding and early prediction of the impacts of such dramatic changes is a challenge for all sciences (including economics, social or medical sciences) but also for our society as a whole [1,2]. Extreme variations in natural processes and phenomena, in many cases enhanced or even caused by human actions, generate hazards that lead to risks for both communities and the environment, and as a result, sometimes disasters occur. The concept of disaster has evolved over time, and here we use an adapted Intergovernmental Panel on Climate Change (IPCC) definition: a disaster is an event, which severely alters the functioning of a community due to hazardous physical, biological or human related impacts leading to widespread adverse effects on multiple scales and systems (environment, economic, social). Immediate emergency response, also as external support, is required for recovery [3]. Disasters are often perceived as acute situations, but they can also be chronic [4]. Most researchers currently view social disruption as the key defining feature or essential dimension of a disaster [5]. The spatial extent of the immediate impact is usually directly related to the physical characteristics of the hazard, but the longer-term effects can encompass larger regions, depending on the functional relevance of the affected areas. For example, earthquakes may cause instantaneous damage and casualties at the sites where they occur, but their adverse consequences for human health, the environment, cultural heritage and economic activity may affect regions and last for years [6].

Pandemics are hazards related to large-scale outbreaks of infectious diseases that can greatly increase morbidity and mortality over a wide geographic area and cause significant economic, social, and political disruption [7]. The consequences of a pandemic, affecting people on a worldwide scale, with expected long-term impacts and consequences on the coupled socio-ecological systems, can be described as a disaster. On 13 March 2020, the World Health Organization (WHO) declared the novel coronavirus disease (COVID-19) a pandemic [8,9] pushing humankind into an ongoing global crisis, which is unique in the recent history, at least by its spatial extent, rapid onset and its complexity of consequences. The COVID-19 pandemic provides substantial challenges to different socio-ecological systems, with clear impacts on many aspects of the environment. It is caused by the Severe Acute Respiratory Syndrome Coronavirus 2 (SARS-CoV-2), and by 29 April 2020, COVID-19 had affected 213 countries, territories and areas across five different WHO regions and three international conveyances [10]. SARS-CoV-2 results in apparently high fatality rates and incapacitated health systems, and the prevention of further transmission has rapidly become a priority [11]. Considering the obvious impacts on all the components of the Earth system, increasing concerns and assumptions related to the changes and consequences following the COVID-19 pandemic are currently under study.

Significant advances were made in technology (e.g., enhanced monitoring, storage and transmission of information, communication systems and efficacy of renewables), medical sciences e.g., novel cancer treatments and remote surgery), environment (monitoring and modelling, e.g., enhanced observational systems and advanced weather and climate models) and biodiversity conservation which has improved the quality of life globally [12]. Despite this significant improvement of quality of life, several potentially destructive issues have dominated the global agenda, such as increasing threats derived from terrorist organisation, climate and environmental changes, nuclear proliferation, economic crises, and the democracy backsliding [13,14,15,16,17,18,19,20,21,22,23]. In this context, the resilience of cities to pandemic disasters has not been properly prepared.

Six major pandemic and epidemic outbreaks swept the planet between 2000 and 2019, namely Severe Acute Respiratory Syndrome (SARS) (2002–2004), H1N1 influenza (2009), Middle East respiratory syndrome (MERS) (2012–2020), the West-African Ebola virus epidemic (2013–2016), the Zika fever (2015–2016) and Avian influenza (2008–2014). None of these, however, achieved the spatial extent and the widespread impacts that the novel coronavirus did.

Significant changes in behaviour are expected and predicting the impact of the pandemic on different sectors is of highly significant societal interest [24]. While [25] provides a review of the medical publications on the Coronavirus disease 2019, this paper aims to present an early overview of the observed and potential consequences of the COVID-19 on the natural and anthropogenic environment with a special focus on cities. Since the scientific investigations and pertinent information about the characteristics and impacts of the pandemic exist only in the formative stages, we present the potential impacts of COVID-19 on the environment in the expectation that future research will pursue these avenues of inquiry. The impact on cities and population health has been immediately noticeable, and this paper includes the most relevant examples available during the development of the events. We argue that the effects of COVID-19 are determined mainly by anthropogenic factors which are becoming obvious as human activity diminishes across the planet [26].

After Introduction (1), this paper is structured in five sections that tackle (2) Methods, (3) COVID-19 and other pandemic events of the 21st century, (4) Impacts on the environment, addressing the physical and ecological systems, environmental affairs at global scale and environmental monitoring and climate services, (5) Impacts on the present and future climate, and (6) Conclusion. Highlights of the impact found on cities and public health are included in all the sections.

## 2. Methods

The main methodologic challenge of this overview was to collect in a very short time relevant evidence related to the environmental impact of COVID-19 pandemic. Various sources have provided abundant information during the time of the pandemic event. Primary literature from the main scientific flux was examined using Thomson Reuters—Web of Science, Scopus and Google Academic. Press releases of international organizations, such as the World Health Organization and the World Meteorological Organization, were valuable sources for official information. Due to the very short period of the development of the event, information provided by online publications was also included in this study but only when they report or link to opinions expressed by validated experts. Multiple checks of the information were performed whenever it was possible.

It worth mentioning the abundance of information available in a particularly short time, which demonstrates the huge interest for the topic. For this investigation, we cite information from 142 bibliographic references (i.e., 54 blogs, 2 books, 2 chapters, 73 peer-review articles and 11 reports), and 116 websites, but we appreciate that we consulted at least a double number of sources.

The collected evidence consists of data and information, observations, viewpoints and studies published during the development of the COVID-19 pandemic, and they were selected for their clear emphasis on environmental issues, urban and public health matters, as argued in the next sections of this study.

## 3. COVID-19 and Other Large-Scale Epidemic Diseases of the 21st Century

The main cause of pandemic events and epidemic diseases is the close interaction between human populations with both domesticated and wildlife pathogens [27]. Most pathogens pass from wildlife reservoirs and enter into human populations through hunting and consumption of wild species, wild animal trade and other contact with wildlife. Urban areas are especially vulnerable through the high population density and mobility.

The COVID-19 dwarfs the six previous large scale epidemics of the 21st century in terms of spatial extent and societal consequences [24], and it is the only pandemic with widespread and complex environmental impacts. We briefly present a few characteristics of the other large-scale epidemic events of the 21st century.

- The Severe Acute Respiratory Syndrome (SARS) occurred in 2003, leading to more than 8000 infections with a mortality rate of approximately 10% and an impact limited only to local and regional economies [28]. The epidemic ended abruptly in July 2003 and no human cases of the SARS coronavirus have been detected since.
- The 2009 H1N1 influenza virus (swine flu) was a pandemic which first appeared in Mexico and the United States in March and April of 2009. It became a global pandemic as a result of global mobility and airline travel and led to an estimated 0.4% case fatality [29].
- Middle East respiratory syndrome (MERS) was first identified in humans in Saudi Arabia and Jordan in 2012 [30]. MERS is considered a zoonotic pathogen, with infected dromedary camels being the animal source of infection to humans [31,32]. By contrast to SARS, which was contained within a year of emerging, MERS continues to have a limited circulation and causes human disease with intermittent sporadic cases, community clusters and nosocomial outbreaks in the Middle East region with a high risk of spreading globally [33].
- The Ebola virus was first detected in 1976 in Zaire (presently the Democratic Republic of Congo). Since the virus was first detected, over 20 known outbreaks of Ebola have been identified in sub-Saharan Africa, mostly in Sudan, Uganda, Democratic Republic of Congo and Gabon [34]. At present, no vaccine or efficient antiviral management strategy exists for Ebola [35]. Although the Ebola virus has substantial epidemic and pandemic potential (due to the ease of international travel), as shown by the 2013–2016 West-African Ebola virus epidemic with approximately 28,000 confirmed cases and 11,000 deaths [36,37], Ebola outbreaks have been geographically limited [34].
- The Zika fever (2015–2016) was first isolated in 1947 from a febrile rhesus macaque monkey in the Zika Forest of Uganda. Since 1954, when the first cases in humans were reported, the Zika virus had only limited sporadic infections in Africa and Asia. However, a large outbreak with approximately 440,000 to 1,300,000 cases spread from Brazil to 29 countries in the Americas in 2015 [38]. In November 2016, WHO announced the end of the Zika outbreak.
- Avian flu (bird flu) was first reported in 1997 in Hong Kong with only 18 infections and 6 human deaths. More than 700 cases of the avian flu have been reported from over 60 countries [39] of the reported outbreaks occurred in 2016 in China [40].

In the absence of any effective treatments, SARS-CoV, MERS-CoV and SARS coronaviruses are of very high societal concern since they could unexpectedly become a global pandemic at any time [30]. As a result, coronaviruses in general have been studied to anticipate their societal and environmental impact. This has immediate application to the COVID-19 virus. Furthermore, [25] summarizes relevant knowledge on the causative agent, pathogenesis and immune responses, epidemiology, diagnosis, treatment and management of the disease, control and prevention strategies of the COVID-19. A calendar of the COVID-19 events potentially related to the environmental impacts is presented as List S1.

**Figure 1:**
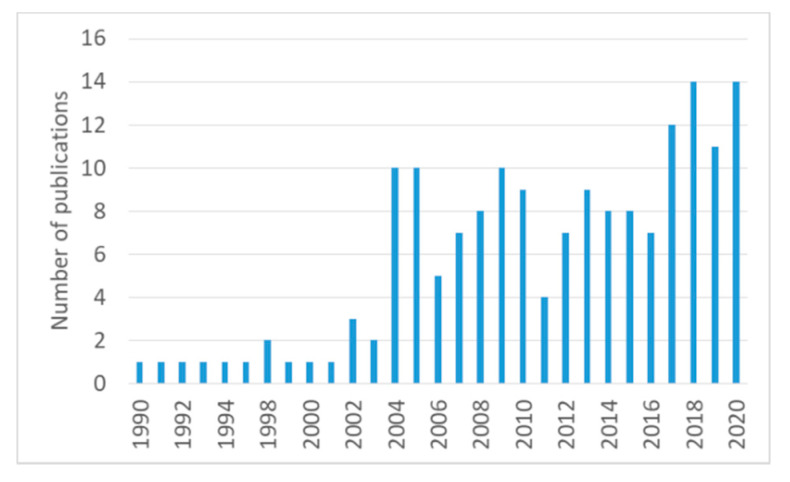
Number of publications referring to the topic coronaviruses and (Climate or Water or Soil or Ecosystems), retrieved on 30 April 2020. The statistics includes studies tackling the bidirectional relation between all types of coronaviruses and the mentioned environmental components. Data source: Web of Science.

The development and spread of COVID-19 under the control of environmental factors justify the scientific interest for the combined studies of coronaviruses on one side and socio-ecological systems (including the interplay between climate, water, soil) on the other side. The number of scientific publications examining such topics has constantly increased in recent decades, and the COVID-19 pandemic strongly motivates the 2020 record (Figure 1).

In general, temperature, humidity, wind and precipitation may favour either the spread or the inhibition of epidemic episodes. However, while some research found that local weather conditions of lowered temperature, mild diurnal temperature range and low humidity may favour the transmission [41], other studies claim there is no evidence that warmer weather can determine the decline of the case counts of COVID-19 [42]. Increased ultraviolet light, as occurs particularly during the summer months, leads to inactivation of the coronaviruses and [43,44] analyse the subject comprehensively and find that warming weather is unlikely to stop the spread of the pandemic.

To understand the relative importance between physical and social parameters that favour the spread of the virus, an area in which different health and social policies have been equally implemented on a variety of environmental and climatic conditions must be examined. Italy is a viable experimental model to examine the impact of different health policies, as stated by the government authorities themselves [45,46]. In Italy, the regionalization of public health has addressed the pandemic following completely different schemes from one region to another and represents an important test to verify the scientific hypotheses on the behaviour of SARS-CoV-2.

Given that coronaviruses tend to spread in lowered temperatures and drier conditions during the winter months (i.e., during a period of reduced solar radiation), it is surprising that Italy was the first European country severely affected by the pandemic and its hospitals were suddenly overrun. Northern Italy experienced a very dry and mild winter caused by the presence of a strong polar vortex. The winter of 2019–2020 was one of the driest winters in 60 years (https://www.arpae.it/dettaglio_notizia.asp?idLivello=32&id=11052). The impact on the social and economic structure of the country immediately gave rise to concerns about the potential transmission pathways of the virus and the spread at European scale.

## 4. Impacts on the Environment

The impact of the COVID-19 pandemic on the environment raised attention from the very beginning of the crisis, consisting of (a) observations and analysis of the immediate effects and (b) estimations related to long-term changes. Qualitative assumptions prevail, while consistent quantitative research must wait for relevant data sets and additional knowledge. Most facets of the environmental impact of the COVID-19 pandemic have not directly resulted from the virus itself. The consequence of abruptly limiting or closing economic sectors, such as heavy industry, transport, or hospitality businesses, has affected the environment directly.

Moreover, the impact of the COVID-19 pandemic on socio-ecological systems may be highly variable, from radical changes in individual lifestyle, society and international affairs [47], to simply facilitating a faster change than would normally have emerged [48]. From an anthropocentric perspective, the pandemic may lead to a more sustainable future, including increased resilience of the socio-ecological systems or shorter supply chains, which is a positive development.

However, it is still possible that some nations will opt for less sustainability by pursuing rapid economic growth and focusing less concern on the environment. While negative impacts on the economy and society in general are probably huge, it is very likely that the global-scale reduction of economic activities due to the COVID-19 crisis triggers a lot of sensible improvements in environmental quality and climatic systems. However, not all the environmental consequences of the crisis have been or will be positive. This includes an increased volume of nonrecyclable waste, the generation of large quantities of organic waste due to diminish agricultural and fishery export levels and difficulties in maintenance and monitoring of natural ecosystems [49].

**Figure 2:**
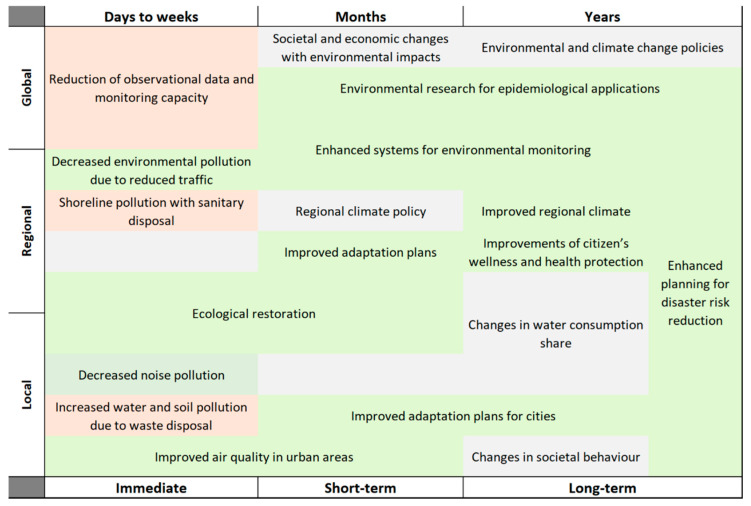
Matrix of observed and potential impacts of COVID-19 on environment and climate change. Red blocks are negative impacts, green are positive and grey stands for neutral effects.

The temporal resolution of the coronavirus impact ranges from immediate (days to weeks), short-term (months) and long-term (years), and different examples are provided in a matrix (Figure 2). While the first impacts are divided between rapid environmental improvements, such as urban air and water quality, and pollution episodes, such as the ones caused by the sanitary disposals, the estimated short- and long-term impacts are mainly positive.

### 4.1. Impacts on the Physical Systems of the Environment

Impacts are rarely limited to a single physical system. However, for the sake of better inventory and understanding, the impact of the COVID-19 on the physical systems focuses on the air, water and soil individually, with an emphasis on urban areas.

Large cities or megacities are often very centralized structures providing a certain degree of comfort and protection for the citizen, but they increase the exposure to specific threats. For example, the higher population densities favour higher exposure to hazards. In contrast to rural areas, where the population tends to have gardens, the effects of the lockdown conditions in cities showed more severe effects on the mental health of individuals living in close quarters. The COVID-19 crisis is driving towards a new paradigm that brings urban policies closer to present and strengthens the future needs of urban population and public health.

One of the key characteristics of the pandemic event in focus in this study is the spatial extent but also versatility of the scale of the impact. No other disaster has covered the whole planet with comparable intensities over so many urban areas with multifaceted threats that are challenging our cities during the crisis.

#### 4.1.1. Air Quality and Local Climate

Air quality is highly sensitive to anthropogenic emissions. In the European Economic Area countries (EU, Norway, Liechtenstein and Iceland), the energy used by industrial processes and the road transportation sector is responsible for about 54% of the nonmethane volatile organic compounds (NMVOC), 51% of the NO_x_, 30% of PM_2.5_ and 25% of SO_x_ emissions [50]. The COVID-19 crisis has caused severe impacts to the energy and resources, high-tech and communications, retail, manufacturing and transportation sectors, in terms of personnel, operations, supply chain and revenue [51]. By mid-April, a 40% to 50% decline in economic activity was estimated as a result of the draconian disease-suppression policies, and severe multiquarter economic impacts in multiple markets became imminent [52]. Consequently, the impact on air quality was rapidly visible at various spatial scales. Even as early as the end of March 2020, reductions in air pollution were reported in China, Italy and New York City, and sharp declines in global greenhouse-gas emissions have been predicted for the rest of the year [53]. Moreover, an overview focused on several European countries reveals that the reduction of the weekly NO_2_, PM_10_ and PM_2.5_ concentrations during March and April 2020 is quasigeneral (Figure S1).

One possible cause of the impact of the pandemic in Northern Italy is that a high concentration of particulate matter (PM, including PM_10_ and PM_2.5_) makes the respiratory system more susceptible to infection and complications of the coronavirus disease. Higher and consistent exposure to PM (particularly for the elderly) leads to a higher probability that the respiratory system is compromised before the onset of the virus. This was a serious concern right after the publication of a position paper by SIMA (Italian Environmental Medical Society), where correlations were found between pollution levels and the spread of the virus [54]. Strong evidence exists on the greater predisposition of the respiratory system to serious diseases [55], but the hypothesis that pollutants can be a carrier for the virus in the free atmosphere seems very unlikely. The spread of droplets produced by sneezing or coughing is necessary so that high viral concentration and a lack of air circulation and exchange can be potentially very dangerous [56,57,58,59].

The analysis of the demographic and economic characteristics of the two Italian regions most affected by the pandemic help to understand that the spread of the virus is dependent on parameters other than simply air transport [60]. The most affected regions are quite similar demographically; Lombardy has a population density of 420 per km^2^ while Veneto has a density of 270 per km^2^ and the average age of the populations is practically identical. Economic indicators also reveal a gross domestic product of Lombardy of 34,000 €/capita and Veneto of 29,500 €/capita. The number of beds in the healthcare facilities for intensive care are nearly identical in the two regions, while there is a public health laboratory for every 3,000,000 inhabitants in Lombardy and for every 500,000 people in Veneto. The healthcare structure is a very important aspect that explains the notable difference between the two regions, as the home care service for the elderly and disabled is more than double in Veneto than in Lombardy [61]. For neighbouring regions with similar pollution levels, the infection rate is extremely uneven. Thus, it appears unlikely that PM is a viable vector for the virus, but it does illustrate the concern over disparate regional healthcare systems. This also has an important impact on future exit strategies from the pandemic and on the use of personal protective equipment (PPE) as the virus may vector using healthcare workers [62].

It is very likely that the Italian case provides lessons for other European countries and validates the measures taken to limit the effects of the pandemic. As for the environmental impacts, physical and ecological systems have been affected in many places, as addressed and detailed in the next sections.

**Figure 3:**
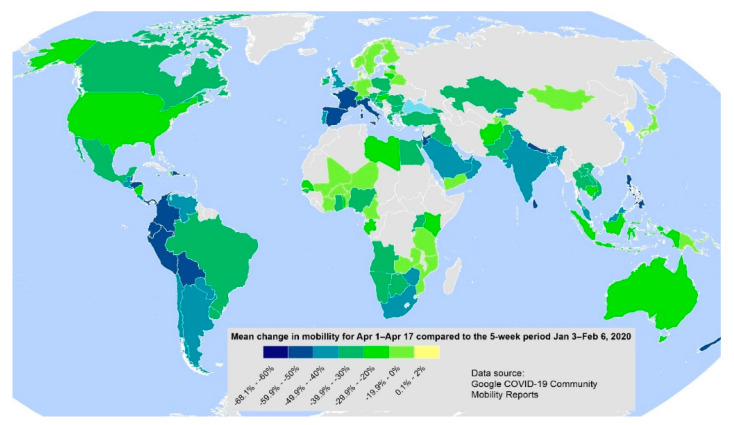
Mean changes in mobility aggregated to country level for the period 1 April –17 April 2020 compared to the 5-week period 3 January–6 February 2020. Countries in grey have no data. Data source: https://www.google.com/covid19/mobility/.

The massive lockdowns of entire cities, economies, schools and social life for weeks led to unknown large-scale and extensive restrictions in mobility as a response to social distancing guidance related to COVID-19 (Figure 3). Globally, largest reductions in mobility are visible for Western and Southern Europe (e.g., Spain—59%, Italy—55%, France—51%) and South America (e.g., Bolivia—60% or Columbia—54%). In South America, mobility in the period April 1 to April 17 showed a mean decrease of 47% compared to the 5-week period 3 January–5 February 2020. Other continents showed a mean decrease of around 30%. South Korea was the only country that showed a slight positive trend of +1.8% for the analysed period. The reason here is that the mobility trends for places like national parks, public beaches, marinas, dog parks, plazas, and public gardens increased significantly, although other mobility categories (e.g., workplaces, transit stations) showed a decrease. Even if general mobility characteristics may vary by country and the period of strongest reductions in mobility may not be evident in April, Figure 3 shows the global picture of the effects of the COVID-19 pandemic.

**Figure 4:**
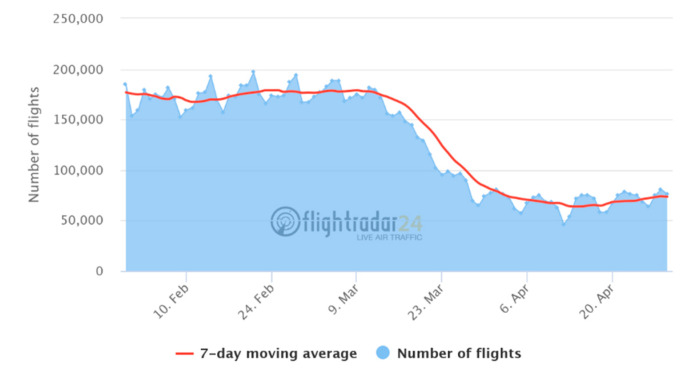
Commercial flights tracked by Flightradar24 between 31 January and 29 April 2020. Source: https://www.flightradar24.com/data/statistics.

In particular, one of the most hit sectors was the aviation that contributes about 1–2% of global greenhouse gas emissions [63] and about 3–5% of global CO_2_ emissions [64]. Between 23 January 2020 and 21 April 2020, travel restrictions caused air traffic to decline by around 63% in the total number of flights and about 75% in the number of commercial flights (Figure 4). The latest scenario of the International Air Transport Association (IATA) suggests that air traffic will fall by 48% for 2020 [65]. Even if the aviation sector returns to its pre-pandemic levels, 40% of the passengers indicate they will wait at least six months before returning to air travel. Specifically, 70% indicate they will wait for their financial situation to stabilize [65]. The strong decrease in both short-term and mid-term aviation travel will lead to a reduction in greenhouse gas emissions, particularly CO_2_. Additionally, the reduction in contrails may increase the daily temperature range [66]. The reduction of contrails will probably lead to a decrease in air temperature due to the decreasing greenhouse effect [67].

**Figure 5:**
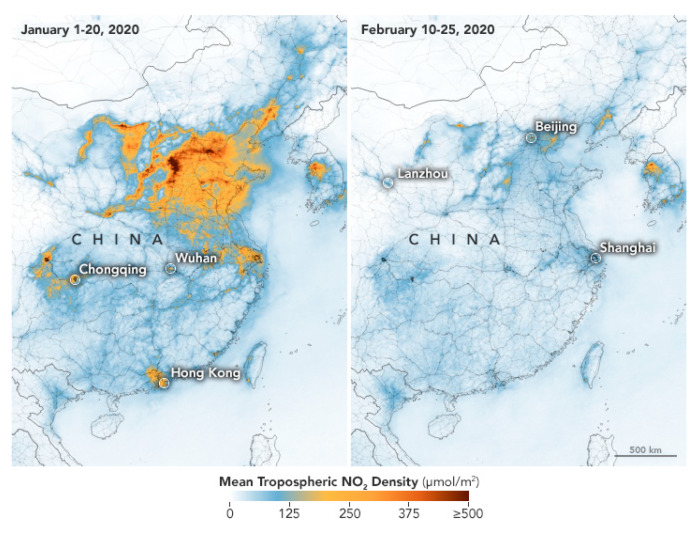
Mean tropospheric NO_2_ values retrieved by NASA Aura and ESA S5TROPOMI pollution monitoring satellites over north-eastern China in January and February 2020. Source: https://earthobservatory.nasa.gov/images/146362/airborne-nitrogen-dioxide-plummets-over-china.

**Figure 6:**
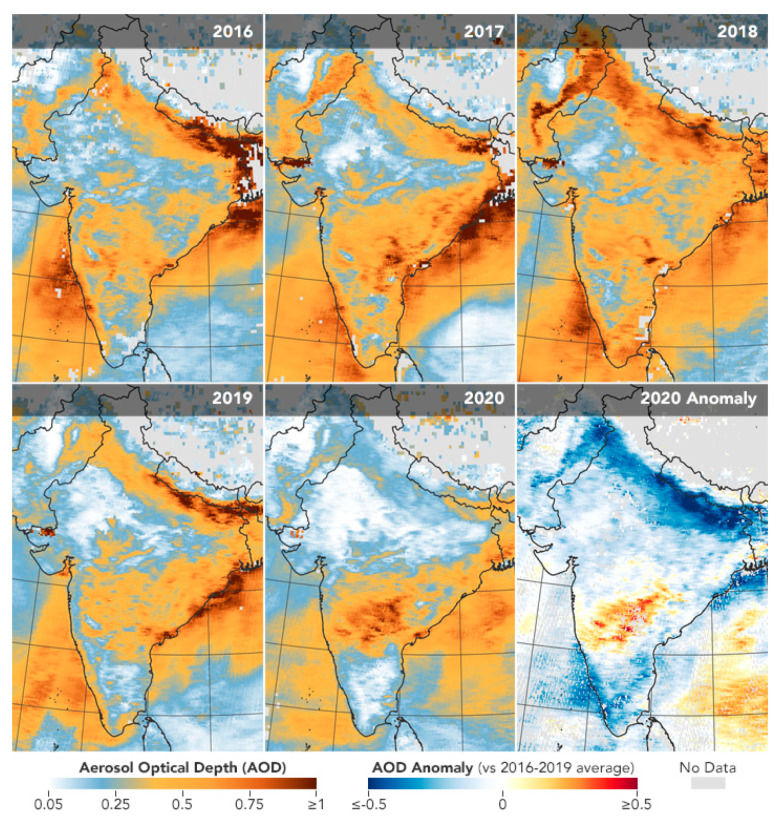
Aerosol optical depth (AOD) over India during March 31 to April 5 from 2016 through 2020. Source: https://earthobservatory.nasa.gov/images/146596/airborne-particle-levels-plummet-in-northern-india.

Satellite remote sensing provides real-time evidence for the beneficial effect of the COVID-19 pandemic on air quality over large areas. For example, both NASA and European Space Agency (ESA) pollution monitoring satellites measured a significant decrease in nitrogen dioxide (NO_2_) over north-eastern China during the economic slowdown of January–February 2020 (Figure 5). Moreover, data retrieved by the Moderate Resolution Imaging Spectroradiometer (MODIS) on NASA’s Terra satellite show that the February–March 2020 lockdown dramatically reduced the aerosol levels in Northern India (Figure 6).

Regarding the transport sector, motor vehicles were responsible for 30% of the 2018 greenhouse gases emissions in Austria, for example. This was the second largest source of greenhouse gas emissions in Austria, behind the energy and industry sectors, which together contributed 36% [68]. The COVID-19 crisis has led to a substantial reduction in motor vehicle traffic with not only a reduction in greenhouse gas emissions and particulate pollution but also a major reduction in traffic noise and tire wear on road surfaces. In Vienna, with a population of 1.9 million, car and truck traffic were reduced by 52% and 50%, respectively, between 1 March and the first week of April [69].

These reductions, extrapolated to similar urban areas in Europe, have led to significantly improved air quality. In Milan, average concentrations of NO_2_ for the 16–22 March period was 21% lower than for the same week in 2019. In Bergamo, average concentrations of NO_2_ in 2020 were 47% lower than in 2019 for the same week, and similar reduction of average NO_2_ concentrations have been observed in other major cities (e.g., Barcelona, 55%; Madrid, 41%; Lisbon, 51%) [70].

Data also show a reduction in the urban PM concentration. Reduced concentrations of PM_2.5_ in Seoul (South Korea) were 54% lower from 26 February to 18 March 2020 when compared to the same period in 2019. Los Angeles (United States) observed its longest continuous period of clean air on record, lasting over 18 days from March 7 to 28. PM_2.5_ concentration levels were lower by 31% from the same time last year and down 51% from the average of the previous four years (https://www.iqair.com/blog/air-quality/report-impact-of-covid-19-on-global-air-quality-earth-day). For Barcelona, [71] reported approximately 50% reduction of NO_2_ and black carbon, 30% decrease of PM_10_ and 33–57% increase of O_3_ concentrations, very likely due to the lockdown of the city. However, the favourable role of meteorological conditions was also granted.

Well-known for its high level of pollution, Milan is considering a shift from car traffic to pedestrian and bicycle over 35 km of streets, as a result of the Coronavirus crisis (https://www.theguardian.com/world/2020/apr/21/milan-seeks-to-prevent-post-crisis-return-of-traffic-pollution). Milan launched on 24 April 2020 a new strategy for adaptation asking for an open contribution from the population [72] where it is clearly stated that the mission is to elaborate a new strategy to exit from pandemic, called Phase 2. The objectives are to remake the city by accounting for problems faced during the pandemic. Public transportation is one of the main foci along with the protections of elderly people.

#### 4.1.2. Aquatic Systems and Water Resources

**Figure 7:**
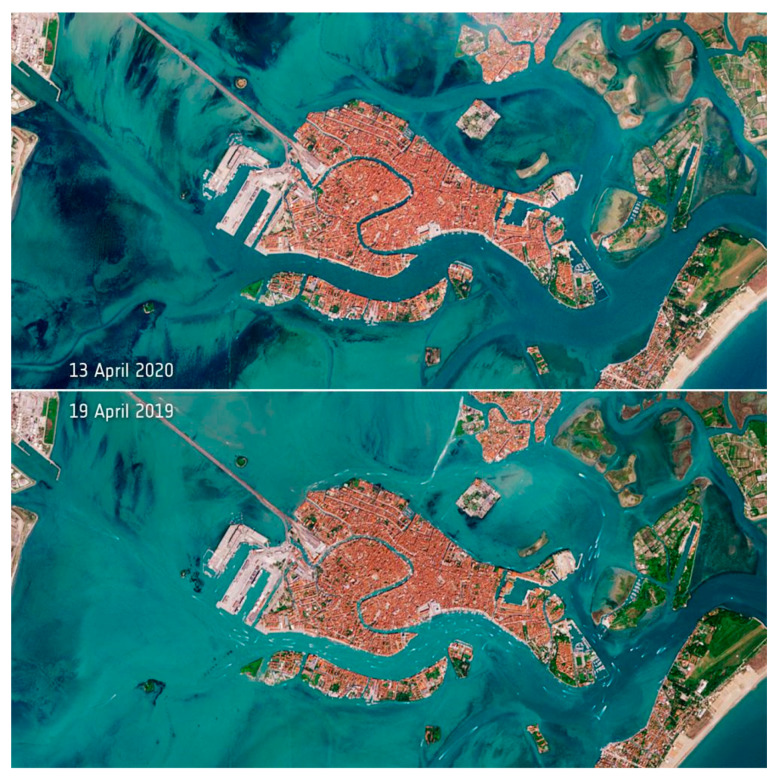
Comparative view of the Venice area between 13 April 2020 vs. 19 April 2019. Satellite images released by the European Space Agency, Copernicus Sentinel-2 mission. Available from http://www.esa.int/ESA_Multimedia/Images/2020/04/Deserted_Venetian_lagoon.

The immediate impact of the COVID-19 pandemic on aquatic systems and water resources is very limited, but water quality and resources may be affected on monthly and annual perspectives. Due to less boat traffic and tourist activities, Venice waters cleared during the Coronavirus lockdown of the city in March and April 2020 (Figure 7).

Reference [73] first detected the presence of the SARS-CoV-2 in sewage and indicated it as a sensitive tool to monitor the circulation of the virus. Although the viral RNA has been detected in wastewater, this does not necessarily imply a risk [74], either to the public or to the environment. Reference [75] showed that coronaviruses die off rapidly in wastewater and are inactivated faster in warmer water (i.e., 10 days in water at 23 °C and >100 days in water at 4 °C).

Disposal of sanitary consumables, such as PPE, is already creating concern about the impact of the pandemic event on water bodies. By May 2020, many reports have claimed significant harm on the aquatic environment especially along the shorelines (e.g., in Hong Kong and Canada) due to sanitary disposal resulting from medical activities or personal protection.

The COVID-19 crisis has and probably will exhibit longer-term impacts on water resources usage and management. The economic effects of the COVID-19 pandemic, changes in national budgets and changes in funding priorities may lead to lack of funding for water related infrastructure and water utilities. The impacts of underfunding (e.g., increased forthcoming losses or lack of investments to improve efficiency) may only manifest after a few years.

During lockdown conditions, water utilities from Germany and Austria report that the daily peak in water consumption in the morning is shifted by around 1.5 to 2 h. Generally, a dampening effect and a more even distribution in water consumption during the day is observed. Regarding the amount of water consumed, increases as well as decreases of around 5% are reported. Increases are explained by higher demands due to watering of gardens—surprisingly, not due to increased hand washing—and decreases by fewer commuters, students and pupils in supply areas [76,77,78,79,80].

By contrast, municipalities with high touristic activity—a leading cause of water demand [81,82]—will exhibit important reduction in water consumption. Reports from the strong tourism heritage of Tirol, Austria, suggest reductions in water consumption of up to 50% in municipalities where tourism plays an important role [79]. Depending on the return of tourism following the end of the pandemic, a noteworthy reduction of water demand and pressures on water resources can be expected.

Industrial water consumption, a generally poorly measured quantity, has certainly decreased. The longer-term impacts on water resources will depend on economic developments following the crisis. In comparison to domestic and industrial water demand, the highest pressures on water resources come from the agricultural sector. Here, long-term forecasts will depend on the return of agriculture following the crisis, although short-term effects are probably visible in reduced irrigation demand.

#### 4.1.3. The Soil Environment

Soil provides essential ecosystem services for human society, ranging from agricultural production to carbon sequestration, which are fundamental for several Sustainable Development Goals (SDGs), such as “Zero Hunger” or “Life on Land” [83]. The immediate impact of the pandemic or other similar disasters on the soil environment is linked with the increasing risks of food insecurity and disruption of the food supply chain.

The persistence of SARS-CoV-2 on different surfaces is a key issue for successfully controlling its spread. Reference [84] found the viruses can remain viable on surfaces for several days. Other studies investigated the survival of different viruses in soils and sediments [85]. At present, there is no clear evidence about the role of the soil environment in hosting and transmission of the SARS-CoV-2 nor about the impact of this coronavirus on the soil surface, and calls for collecting eventual results have been issued [86].

### 4.2. Impacts on the Ecological Systems

From an ecological perspective, the COVID-19 crisis is fundamentally related to the relationships between society and ecosphere. While the origin in a Wuhan wet market or industrial livestock or other source is not yet fully clarified [87], it is well known that MERS-CoV, SARS-CoV and SARS-CoV-2 are all animal coronaviruses which infected people and then succeeded to spread in different communities at large scale. Around the globe more than 2.7 million people are dying from zoonosis in a year [88], but the impact is even greater as the zoonosis are also affecting human health, livestock sector and agriculture and usually the poorer human populations are more affected.

The Coronavirus crisis is most probably one of the many challenges our society will have to face in the forthcoming decades as an indirect consequence of the impact of climate change on the ecosphere through many mechanisms, including diminishing species habitats [89], changing species distributions [90] and an increasing influx of alien invasive species [91]. Currently, economic development focuses on continuous growth without considering the conservation of natural systems.

In a letter sent to the WHO (World Health Organisation) in April 2020, more than 300 animal welfare and conservation organisations stressed the need to recognise the link between wildlife markets and pandemics (https://lioncoalition.org/2020/04/04/open-letter-to-world-health-organisation/). However, this is related with the need to act on existing international conventions, such as CITES (the Convention on International Trade in Endangered Species of Wild Fauna and Flora, also known as the Washington Convention) to protect endangered plants and animals from trafficking. As this is not the first time such outbreaks have occurred (see the SARS event between 2002 and 2003), conventions like CITES should be reinforced. Forest landscape fragmentation also may facilitate more often human contact with wild animals, increasing the likelihood of transmission risk of animal-to-human viruses [92,93].

The pandemic has also had an impact on ecological research, field work and experiments. In many cases, this research activity has been diminished or halted, with important consequences on conservation of species and habitats. There is also a possible economic impact on conservation programs around the globe as a result of pandemic and different programs are assessing their long-term viability (such as the Global Environmental Fund) [89]. Even after the pandemic ends, a danger exists that both research and conservation programs will be diminished mainly due to miscommunication between decision makers and scientists.

**Figure 8:**
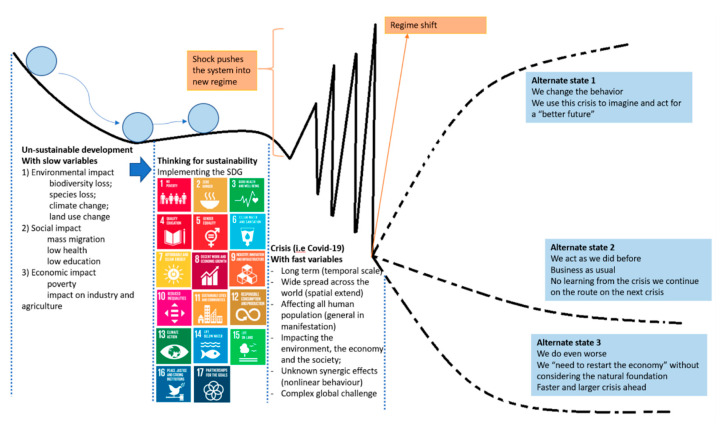
Possible regime shifts due to the combined impact of slow and fast variable on the system dynamics.

However, perhaps the most important impact of the pandemic on the ecological transition focuses on sustainability and the still possible choices that the society could make to ensure its long-term survivability. As explained in Figure 8, the coupled natural-human system is on a path of transitioning from an unsustainable development towards sustainability being under pressure from different drivers. The instability caused by the pandemic is characterized by variables that have sudden and multiple impacts on both the natural environment and on society and could push the system into three different potential states. The fast variables are characterizing the instability phase and the slow variables act as controlling variables [94,95,96]. Only one of these potential states is the desirable one, moving away from unwanted events and ensuring that the pandemic was a painful but still a “learning event” that drove towards a “better future”. The main characteristic of the pandemic is that it is acting like a shock that pushes the system towards a regime shift with difficult to predict consequences.

Reference [93] advocates that nature is part of the solution for recovery and sustainable reconstruction. Nevertheless, the effect of the COVID-19 pandemic on ecological systems has not yet been fully realized, and further monitoring will bring new findings and perspectives.

### 4.3. Impacts on Environmental Dimension of the Global Affairs

It is very likely that the COVID-19 pandemic will reshape the economic and environmental policies at an international scale.

The strength of some bilateral agreements and international partnerships has been tested by this pandemic. Whereas China persistently invested in Africa’s natural resources and infrastructure projects, the treatment of African citizens living in China and the frustration at Beijing’s opposition on granting debt relief could deteriorate the Chinese economic and political supremacy in Africa [97]. Reference [98] also discusses the impact of the crisis on African economies with unpredictable environmental consequences. The roles that China and the USA currently play for mitigating risk include an ecological emphasis to the pandemic strategy preparedness in order to better protect the global community from zoonotic disease [99]. The Coronavirus epidemic could significantly impact the Italians’ relationship with the EU, as indicated by the widely spread perception that the EU was not efficient in supporting the fight against Coronavirus [100], at least in February–March (i.e., 88% of Italians believed so in March 2020). Such changes are expected to generate indirect long-term environmental impacts.

Climate changes are often perceived as a risk driver at the global scale and COVID-19 has offered an excellent example of how a single underestimated threat can challenge the foundations of global security, economic stability and democratic governance [101]. According to analyses before the COVID-19 pandemic, if countries are unable to implement the nationally determined contributions as ratified through the Paris Agreement, the emissions reduction efforts would cost the whole world about 149.8–792.0 trillion dollars until 2100 [102]. Plans prepared for reinforcing the emission reduction goals established under the 2015 Paris Agreement are not only postponed until 2021, but they will probably suffer consistent adjustments in the new economic circumstances. In the short term, it is hard to assume that climate change and environmental sustainability will be priorities for the world governments or local authorities, while the long-term cost for emission reduction could be raised. The Coronavirus crisis also threatens local commitments to implement climate change adaptation and mitigation measures that have been initiated in the recent period [103]. Both national and international governance will be affected.

The impact of Coronavirus on the EU climate plan was already the subject of discussions in several meetings in Brussels and there are concerns that the targets set for 2050 now will be difficult to reach especially due to the necessity for a rapid economic recovery. Poland, in particular, expressed doubts on reaching the targets set for 2050 (http://www.caneurope.org/publications/press-releases/1864-eu-aims-for-net-zero-emissions-by-2050-now-it-needs-to-work-on-raising-the-2030-target). Big industries such as car manufacturers also have expressed concerns of not being able to meet the targets set (https://www.carbonbrief.org/daily-brief/eu-leaders-agree-to-consider-climate-in-coronavirus-recovery-plan).

During the 2010s, environmental efforts have intensively addressed the generous framework of the “Transforming our World: the 2030 Agenda for Sustainable Development” [104]. This agenda includes 17 Sustainable Development Goals (SDGs) designed to eradicate poverty and achieve sustainable development by 2030. We argue that most of these goals were immediately impacted by the COVID-19 pandemic, while longer-term effects are also expected (Table 1), most of them directly connected to urban areas and population health. It is very likely that the concept and implementation of the agenda must be reconsidered according to the new findings related to our exposure, vulnerabilities and resilience to global disaster risks.

### 4.4. Environmental Monitoring and Climate Services

The COVID-19 crisis has challenged environmental monitoring and climate services, creating both adversities in observations as well as challenges to create better preparedness.

Lack of reliable data on the spread of COVID-19 could lead to not only a once-in-a-century pandemic but also a once-in-a-century decision fiasco [110]. The crisis has revealed the crucial need to access long-term, real-time data for supporting policy makers and reaction at different scales, and it has motivated environmental scientists to reinforce our monitoring capacity to address sustainability issues the pandemic has raised [121].

Challenges like the dearth of airborne meteorological measurements or the maintenance of environmental monitoring in protected areas will gradually be resolved once previous levels of social and economic activity resume [49]. However, actions are needed now to build reliable responses to future threats.

**Figure 9:**
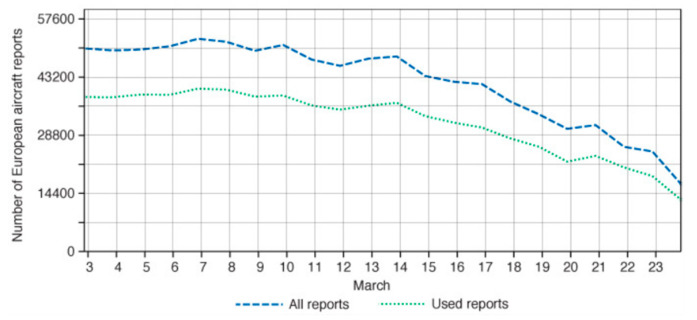
Number of daily aircraft reports over Europe received and used at ECMWF. Source: https://www.ecmwf.int/en/about/media-centre/news/2020/drop-aircraft-observations-could-have-impact-weather-forecasts.

The COVID-19 crisis has strongly biased the production and delivery of both weather forecasting (https://news.un.org/en/story/2020/04/1060772) and climate services (i.e., climate-based information and products tailored for various end-users related to the present climate and adaptation to different scenarios) as well as the observation of oceans and remote locations (https://www.theguardian.com/science/2020/apr/03/climate-monitoring-research-coronavirus-scientists#maincontent). The pandemic has dramatically lowered the quantity and quality of aircraft weather observations, thereby adversely impacting weather forecasts and modelling efforts. The European Centre for Medium-Range Weather Forecasts (ECMWF) has noted a reduction of 65% in aircraft reports received between 3 March and 23 March (Figure 9). On 9 April, the World Meteorological Organization (WMO) issued its concern about the impact of the crisis on the Global Observing System [122].

However, the exceptional slowdown of societal activities that began in March of 2020 has generated opportunities to capture environmental information of a novel event. For example, the “noise” associated with human activities that adversely affect seismographic records dropped sharply around the world, improving the ability to detect seismic waves and the locations of earthquake aftershocks [123].

## 5. Impacts on the Present Climate and Climate Change

Transmission of diseases by population mobility within the context of climate change received scientists’ attention before the current pandemic [124,125].

The examination of the relationship between climate and Coronavirus focuses on two queries: (a) how the climate can modulate the spread and persistence of the virus, and (b) the extent of the impact of the virus on economic policies taken to offset climate impacts. The first aspect is inherently scientific and mainly involves the atmospheric and epidemiological disciplines. The second is much more complex as the economic, political and social dynamics will affect processes that will alter our worldview.

Climatic effects on the Coronavirus are currently difficult to estimate given that this pandemic is still under development. These effects, therefore, can only be speculated by comparing them to the characteristics of other coronaviruses. Reference [118] investigated the observed growth rate of Coronavirus worldwide and related it to the climate, making a prediction for forthcoming seasons. They argue a specific climate exists in which the Coronavirus spreads optimally. Outbreak dynamics also were investigated in terms of climate and environmental conditions [126] to link directly daily growth rates to the local climate. The correlation found was significant leading them to conclude that such a link was valid, but their study also highlighted the fact that population density could be a confounding variable. These results, although very speculative, have led to initial hypotheses on the transmission conditions of SARS-CoV-2 under different combinations of atmospheric parameters [127] and to forecast conditions for the summer of 2020 [128,129]. An analogy with the other Coronaviruses becomes fundamental to validate such hypotheses but it is not currently possible to establish whether the virologic characteristics of the new pathogen can be assumed to be like other coronaviruses.

Analysing the direct and indirect effects of the pandemic on the climate is more complicated as forecasts must resolve not just the contagion dynamics but also incorporate economic, social, and political aspects of the virus propagation.

**Figure 10:**
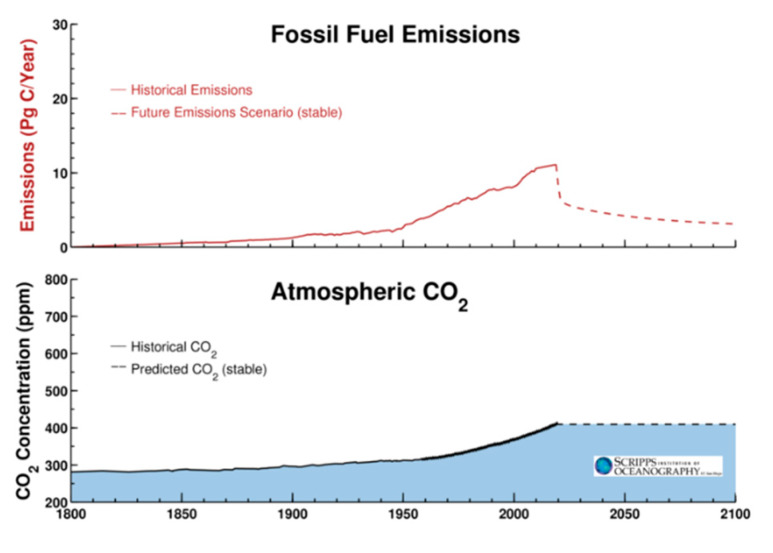
Emission trend for RCP6 scenario (Source: The Scripps Research Institute, Available from https://scripps.ucsd.edu/programs/keelingcurve/).

Direct effects on climate change could result mainly from the global slowdown of production activities and transportation. At this stage, the overall effects are not easily determined but, for example, emissions in China—the country with the longest period of closure—have decreased by 25% [130], corresponding to a decrease of about 200 million tons of CO_2_ in February alone [131]. Nevertheless, the possible decrease in global CO_2_ emissions is likely to be around 5% worldwide [132] (Reuters, 2020). For the Representative Concentration Pathway (RCP)6 climate change scenario, Scripps Research Institute [20] suggests a possible trend in emissions (Figure 10) which shows an immediate drop followed by a recovery when activities resume.

This projection leads to fundamental speculations as to what indirect effects Coronavirus will have on the Earth’s climate. We note that following the 2008–2009 economic crisis, CO_2_ emissions exhibited rapid growth [133] and we suggest that a similar response will follow this pandemic.

Experts suggest one of two sharply divergent paths will arise from the demise of the pandemic [134]. On the one hand, a feeling exists that the Coronavirus will support the government, science, and business infrastructure in addressing environmental issues, including climate change [135]. Although the Coronavirus and climate change operate on different time scales, they represent similar phenomena in terms of the evolution and impacts of the problem. Thus, lessons from the pandemic provide lessons to be learned in environmental protection. Recovery from the pandemic, therefore, may lead the focus away from environmental concerns [53].

Surely something has already changed. COVID-19 has undermined the basic tenets of global manufacturing. Companies must now reconsider the multistep, multi-country supply chains that dominated production and derivative production [132]. Individuals too must reconsider life choices as profound changes also await us [131,136].

## 6. Conclusions

The COVID-19 pandemic has triggered unprecedented environmental impacts in terms of spatial extent, complexity and even uniqueness. It is the first time in history that the metabolism of all the urban agglomerations with more than 1 million inhabitants from Europe was virtually stopped regarding movement, traffic and economic exchanges. The societal and economic measures adopted to contain the pandemic led to local, regional and global impacts, both negative and positive, spanning from immediate to long-term consequences. The full evaluation of the impacts is far from being possible with an ongoing disaster of epic proportion and tremendous complexity, and this paper pledges for several directions to be pursued by further research.

The COVID-19 pandemic provides a clear demonstration that human and planetary health are intimately interconnected [137], and the role of interdisciplinary approaches in finding solutions has been clearly highlighted [138]. The disaster reached the planetary scale within only two months (i.e., February through March 2020). Despite six other pandemic outbreaks having occurred during the 21st century, humankind was still not prepared to deal with a global event. Most countries adopted a strict lockdown of economies and societal activities, triggering immediate impacts on many physical and ecological systems. Longer-term consequences are also assumed, and a systemic approach is required to support the prevention, early warning, and similar impacts of environmental degradation.

The Coronavirus pandemic has generated an active involvement of the research community and has garnered an early response from international, national, and local authorities. Since the events are ongoing and the end is still difficult to predict, we shall refer only to preliminary results and possible lessons to be learned. The reaction of the scientific community to the crisis was prompt and led to rapid accumulation of knowledge and operational decisions. Faced with an unprecedented interruption of data from aeronautical meteorological service providers (AMSPs) and other observational platforms, the WMO has enumerated preliminary guidelines to assist the AMSPs [122] at the beginning of April 2020. Eventually, problems associated with environmental monitoring have reinforced the need to secure backup systems to collect information, as such data are crucial for operational forecasting of ecological, weather and hydrological conditions. Of note, relationships between weather conditions and the spread of the virus are still unclear and more research is needed to derive relevant conclusions.

The advancements of new specific techniques would be of great interest for controlling the environmental dissemination of coronaviruses [126], and more precise and extended monitoring would favour the collection of more relevant information. Early developments with this crisis have revealed that monitoring of socio-ecological conditions is crucial for an early intervention to limit the scale of the epidemic and the pandemic hazard. Reference [139] argues that better monitoring of immigrant tracks and travel volumes could have helped countries be better prepared to contain the spread of the novel coronavirus. Data, tools and lessons learned may provide significant improvements in preparation to fight potential pandemics in the future [140].

This global crisis has convincingly demonstrated that the disaster research, climate change diplomacy and ecosystem services must reconsider their strategic and integrated development considering even the most unlikely events. Eventually, the COVID-19 pandemic will determine profound changes of the social and economic behaviour at the planetary scale, and this study highlights the environmental dimension of the consequent impacts resulting from the emerging pandemic.

## Figures and Tables

**Table 1:** Observed and estimated impacts of the COVID-19 pandemic on the Sustainable Development Goals (SDGs).

SDGs	COVID-19 Observed and Potential Impacts (Examples)	Time Scales of the Impact	References
1: No Poverty	Deep economic and financial crisis could dramatically increase extreme poverty.	Days to decades	[98]
2: Zero Hunger	The crisis has caused a massive slowdown of the efforts to support investment, including through enhanced international cooperation. Impact on the Water, Energy and Food security nexus.	Days to decades	[2]
3: Good Health and Well-being	Greater attention to the elderly and the fragile population for the growth of dedicated assistance services and access to medical and food resources.	Days to decades	[105]
4: Quality Education	Education systems were forced to abruptly change procedures, shifting from physical to online teaching.	Days to decades	[106]
5: Gender Equality	Pre-existing inequalities in the labour market have been deepened.	Days to years	[107,108]
6: Clean Water and Sanitation	The discovery of the permanence of the virus on surfaces and in aquifers requires a revision of the purification and sanitation systems.	Days to years	[109]
7: Affordable and Clean Energy	Alternative energy sources and backup storage and transport systems should be developed to secure societal needs during crises.	Years to decades	[110]
8: Decent Work and Economic Growth	The pandemic has shown that there are groups of workers most exposed to risk to health and life by requiring a revision of the working methods in industry, commerce and health.	Months to decades	[105,111]
9: Industry, Innovation and Infrastructure	Technological innovation and a close link with the research invention, also to the advantage of a change in production methods, has proved to be an unavoidable condition for the solution of global problems	Months to decades	[112,113]
10: Reduced Inequality	Improvements in access to information technologies to reduce inequalities in poor and large families who have to use remote school systems and access to other resources.	Days to months	[24,114]
11: Sustainable Cities and Communities	Revisions of adaptation plans are foreseen for major cities to increase health resilience in citizens and to better protect elderly population.	Months to decades	[72,115,116]
12: Responsible Consumption and Production	Revision of production systems from the global to the local scale to ensure access and distribution of strategic resources with consequent enhancement of territorial activities.	Months to decades	[117]
13: Climate Action	Transfer of the concepts learned from pandemic evolution to the climate issue.	Months to decades	[118]
14: Life Below Water	No evidence of significant observed or potential impact.	Not the case	[119]
15: Life on Land	Possible financial shortages on protected areas, Increase microplastics in water and soil; Strong support for closing the wildlife trade; Concerns about the influence of habitat loss on epidemic episodes.	Months to decades	[92,93]
16: Peace and Justice Strong Institutions	The importance of strong coordination between institutions has been markedly indicated for national ones but, above all, for international ones where the exchange of exact and punctual information can indicate safe ways for solving problems on a global level.	Months to decades	[99,120]
17: Partnerships to achieve the Goal	The efficiency of international agreements have been dramatically challenged, and the need for rethinking regional and global partnerships emerged.	Days to decades	[103]

## Supplementary Materials

The following are available online at https://www.mdpi.com/1660-4601/17/11/4140/s1, List S1: Calendar of events relevant for COVID-19 impact on environment (31 December 2019–30 April 2020), Figure S1: Average weekly NO_2_ concentration between January 2019 and April 2020 in urban areas from Spain, France, United Kingdom, Italy, Austria and Poland. Source: https://www.eea.europa.eu/themes/air/air-quality-and-covid19/monitoring-covid-19-impacts-on.
