# Supplementary material for: Observed and Potential Impacts of the COVID-19 Pandemic on the Environment
Source: Int J Environ Res Public Health. 2020 Jun 10;17(11):4140. doi: 10.3390/ijerph17114140 (PMC7311982; doi:10.3390/ijerph17114140)
Supplement: Supplementary file 1 [file ijerph-17-04140-s001.zip › Supplementary_01.docx]

**Supplementary Material 1:**

Calendar of events relevant for COVID-19 impact on environment (31 December 2019-30 April 2020)

31 December 2019

A pneumonia of unknown cause detected in Wuhan, China was firstly reported to WHO

13 January 2020

First case of novel coronavirus outside of China confirmed

24 January 2020

Wuhan’s lockdown directives on its 11 million residents resulted in the city experiencing its cleanest air quality on record for the months of February and March. (COVID-19 AIR QUALITY REPORT, 2020)

30 January 2020

The outbreak was declared a Public Health Emergency of International Concern

19 February 2020

An analysis made by Lauri Myllyvirta (Centre for Research on Energy and Clean Air) covering air quality and energy trends in China showed that Coronavirus temporarily reduced China’s CO2 emissions by a quarter.

<https://www.carbonbrief.org/analysis-coronavirus-has-temporarily-reduced-chinas-co2-emissions-by-a-quarter>

28 February 2020

NASA and European Space Agency (ESA) pollution monitoring satellites have detected significant decreases in nitrogen dioxide (NO2) over China.

<https://earthobservatory.nasa.gov/blogs/earthmatters/2020/03/05/how-the-coronavirus-is-and-is-not-affecting-the-environment/>

11 March 2020

WHO characterizes COVID-19 as a pandemic expressing as well concerns by the alarming levels of inaction

13 March 2020

With more reported cases and deaths than anywhere in the world, Europe becomes epicentre of the pandemic

13 March 2020

ESA announced that new data from the Copernicus Sentinel-5P satellite reveal the decline of air pollution, specifically nitrogen dioxide concentrations, over Italy.

<https://www.esa.int/ESA_Multimedia/Videos/2020/03/Coronavirus_nitrogen_dioxide_emissions_drop_over_Italy>

17 March 2020

France goes into lockdown

23 March 2020

Germany goes into lockdown

24 March 2020

India goes into lockdown

25 March 2020

The European Environment Agency’s (EEA) data confirm large decreases in air pollutant concentrations due to reduced traffic and other activities, especially in major cities under lockdown measures.

<https://www.eea.europa.eu/highlights/air-pollution-goes-down-as>

25 March – 11 April 2020

India, the world’s largest lockdown had a dramatic impact on the improvement of air quality.

31 March 2020

The World Wide Fund for Nature (WWF) highlights that the loss of nature facilitate the spread of diseases, including new strains of bacteria and viruses, and increases the risk of pandemics.

<https://wwf.panda.org/?361716>

2 April 2020

The number of confirmed COVID-19 cases around the world reached 1 million

6 April 2020

Unite Nation Environmental Programme (UNEP) statement on COVID-19 declares that is ready to support Member States and frontline UN partners in providing technical expertise on chemicals and hazardous waste management.

<https://www.unenvironment.org/news-and-stories/statement/unep-statement-covid-19>

April 6 2020

As a result of rapid air quality improvement during the lockdown in European major cities the European Environment Agency has developed a viewer that tracks the weekly average concentrations of nitrogen dioxide (NO2) and particulate matter (PM10 and PM2.5). (<https://www.eea.europa.eu/themes/air/air-quality-and-covid19/monitoring-covid-19-impacts-on>)

8 April 2020

Wuhan, in Hubei Province - lifted its lockdown

9 April 2020

The World Meteorological Organization (WMO) stated its concern about the impact on the Global Observing System

<https://public.wmo.int/en/resources/coronavirus-covid-19/impacts-global-observing-system>

10 April 2020

WMO emitted a preliminary guidelines for aeronautical meteorological service providers (AMSPs) during the coronavirus (COVID-19) pandemic.

13-15 April 2020

Europe eases lockdown

20 April 2020

Lockdown restrictions ease further

28 April 2020

European Commission President Ursula von der Leyen promises to put the European Green Deal at the centre of the EU’s recovery plan

<https://www.euractiv.com/section/energy-environment/news/green-deal-will-be-our-motor-for-the-recovery-von-der-leyen-says/>

21 April 2020

In the context of COVID-19 pandemic OECD releases a document in which highlights the importance of environmental health through better air quality, water and sanitation, waste management which reduce the vulnerability of communities to pandemics and improve overall societal well-being and resilience.

<https://read.oecd-ilibrary.org/view/?ref=129_129937-jm4ul2jun9&title=Environmental-health-and-strengthening-resilience-to-pandemics>
