# Supplementary material for: Observed and Potential Impacts of the COVID-19 Pandemic on the Environment
Source: Int J Environ Res Public Health. 2020 Jun 10;17(11):4140. doi: 10.3390/ijerph17114140 (PMC7311982; doi:10.3390/ijerph17114140)

**Supplementary material.**

Figure 1. Average weekly NO_2_ concentration between January 2019 and April 2020 in urban areas from Spain, France, United Kingdom, Italy, Austria and Poland. Source: https://www.eea.europa.eu/themes/air/air-quality-and-covid19/monitoring-covid-19-impacts-on


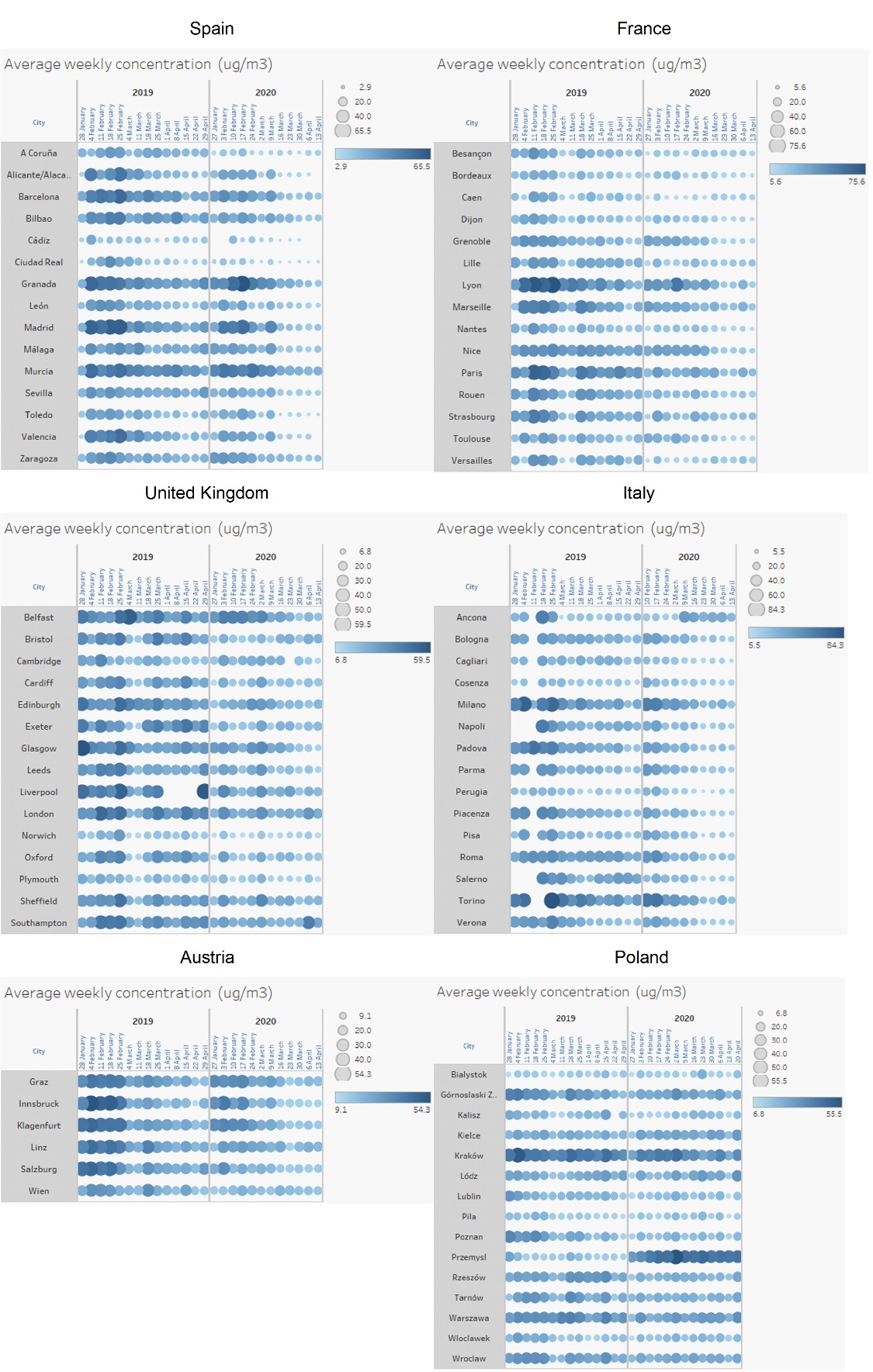


Figure 2. Average weekly PM10 concentration between January 2019 and April 2020 in urban areas from Spain, France, United Kingdom, Italy, Austria and Poland. Source: https://www.eea.europa.eu/themes/air/air-quality-and-covid19/monitoring-covid-19-impacts-on


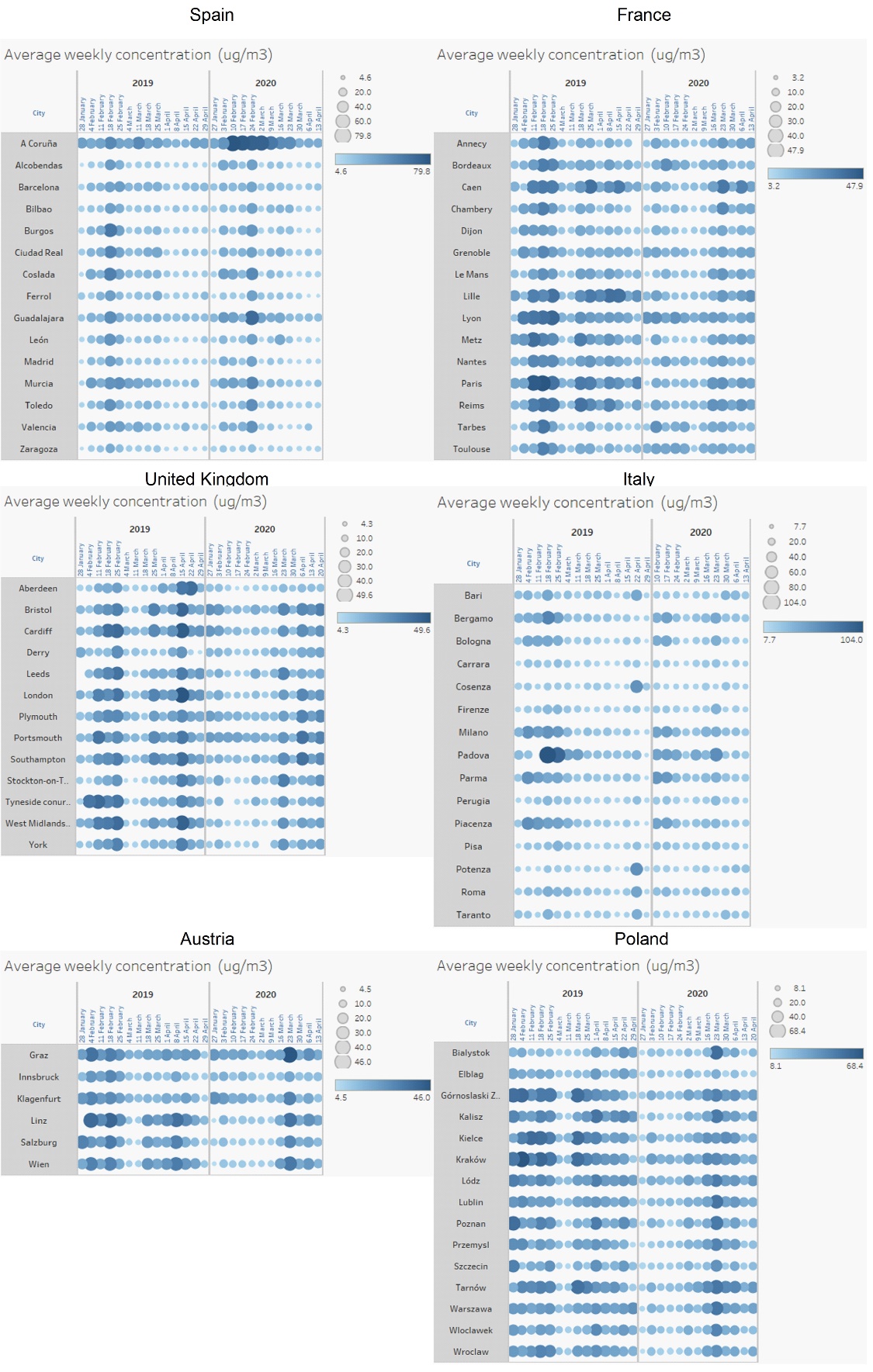


Figure 3. Average weekly PM2.5 concentration between January 2019 and April 2020 in urban areas from Spain, France, United Kingdom, Italy, Austria and Poland. Source: https://www.eea.europa.eu/themes/air/air-quality-and-covid19/monitoring-covid-19-impacts-on


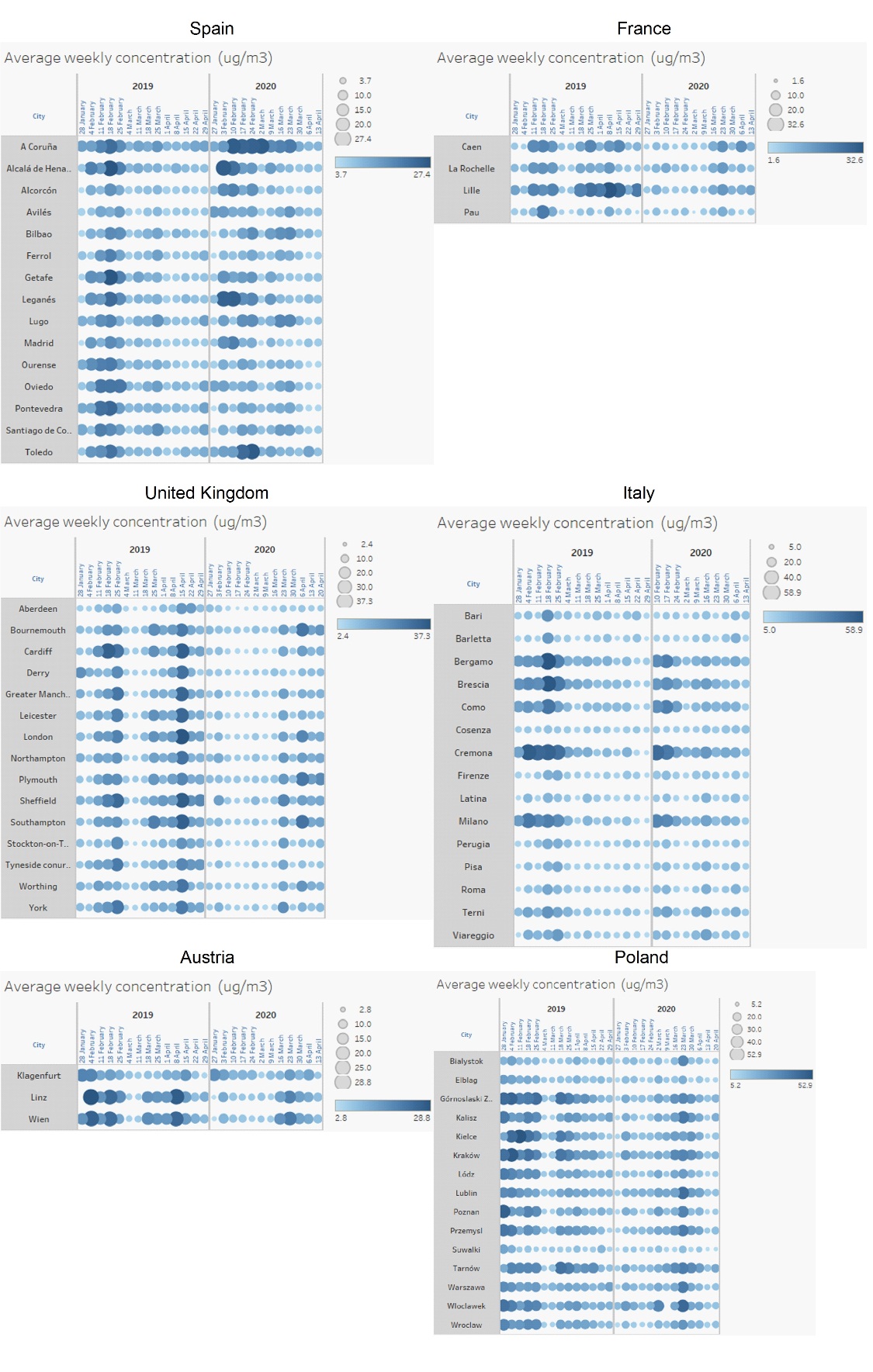

Supplement: Supplementary file 1 [file ijerph-17-04140-s001.zip › Supplementary_02.docx]
